# Bil2 Is a Novel Inhibitor of the Yeast Formin Bnr1 Required for Proper Actin Cable Organization and Polarized Secretion

**DOI:** 10.3389/fcell.2021.634587

**Published:** 2021-02-09

**Authors:** Thomas J. Rands, Bruce L. Goode

**Affiliations:** Department of Biology, Rosenstiel Basic Medical Sciences Research Center, Brandeis University, Waltham, MA, United States

**Keywords:** actin, cable, formin, secretion, Bud6, Bni1, Bnr1, Bil2

## Abstract

Cell growth in budding yeast depends on rapid and on-going assembly and turnover of polarized actin cables, which direct intracellular transport of post-Golgi vesicles to the bud tip. *Saccharomyces cerevisiae* actin cables are polymerized by two formins, Bni1 and Bnr1. Bni1 assembles cables in the bud, while Bnr1 is anchored to the bud neck and assembles cables that specifically extend filling the mother cell. Here, we report a formin regulatory role for YGL015c, a previously uncharacterized open reading frame, which we have named *Bud6 Interacting Ligand 2* (*BIL2*). *bil2*Δ cells display defects in actin cable architecture and partially-impaired secretory vesicle transport. Bil2 inhibits Bnr1-mediated actin filament nucleation *in vitro*, yet has no effect on the rate of Bnr1-mediated filament elongation. This activity profile for Bil2 resembles that of another yeast formin regulator, the F-BAR protein Hof1, and we find that *bil2Δ* with *hof1Δ* are synthetic lethal. Unlike Hof1, which localizes exclusively to the bud neck, GFP-Bil2 localizes to the cytosol, secretory vesicles, and sites of polarized cell growth. Further, we provide evidence that Hof1 and Bil2 inhibitory effects on Bnr1 are overcome by distinct mechanisms. Together, our results suggest that Bil2 and Hof1 perform distinct yet genetically complementary roles in inhibiting the actin nucleation activity of Bnr1 to control actin cable assembly and polarized secretion.

## Introduction

Formins are a conserved family of actin assembly-promoting proteins that perform essential roles in numerous actin-based cellular and physiological processes (Kovar, 2006; Chhabra and Higgs, 2007; Chesarone et al., 2010). Formins nucleate the assembly of new actin filaments and accelerate actin filament elongation while protecting growing barbed ends from capping proteins (Goode and Eck, 2007; Breitsprecher and Goode, 2013). Studies in yeast have led to important advances in our understanding of formin activities, mechanism, and regulation (Breitsprecher and Goode, 2013). Mammalian genomes encode 15 different formins (Higgs and Peterson, 2005), which are used to assemble a wide variety of cellular actin structures, including filopodia, stress fibers, stereocilia, and lamellipodia (Faix and Grosse, 2006). In contrast, the budding yeast *Saccharomyces cerevisiae* has just two formin genes, *BNI1* and *BNR1*, and during most stages of the cell cycle they assemble only a single actin structure, the actin cable network. The relative simplicity of the yeast system, combined with its genetic tractability, have made it a powerful model for elucidating formin regulatory mechanisms.

Bni1 and Bnr1 assemble actin cables from distinct locations in cells, the bud cortex and bud neck, respectively (Moseley and Goode, 2006). However, together they share one essential function in actin cable network assembly, which is crucial for secretory vesicle transport and polarized cell growth (Imamura et al., 1997; Evangelista et al., 2002; Sagot et al., 2002a; Gao and Bretscher, 2008). Bni1 is transiently recruited from the cytosol to the bud cortex, where it assembles cables that fill the bud compartment and extend into the mother cell. In contrast, Bnr1 is stably anchored to the bud neck, where it assembles cables that specifically extend into the mother cell (Ozaki-Kuroda et al., 2001; Pruyne et al., 2004; Buttery et al., 2007, 2012; Gao et al., 2010). Actin cables extend toward the back of the mother cell at a rate of ∼0.3–0.7 μm/sec, and are turned over at the same rate, which means that Bni1 and Bnr1 continuously nucleating and elongating cables (Yang and Pon, 2002; Yu et al., 2011). Post-Golgi secretory vesicles are transported along the cables toward the bud tip by the essential myosin V protein (Myo2) (Govindan et al., 1995; Pruyne et al., 1998). It is believed that the dynamic and constant assembly of the actin cable networks enables them to be rapidly rearranged, and for cells to reorient polarized secretion and cell growth during different stages of the cell cycle (Bi and Park, 2012) and in response to external stimuli, e.g., during the mating response (Ghose and Lew, 2020) and wound healing (Kono et al., 2012). In addition, the rearward (or ‘retrograde’) treadmilling of the cables provides a quality control mechanism to help keep damaged organelles and proteins, accumulated during cellular aging, out of the bud and increase the fitness of daughter cells (Higuchi et al., 2013). However, these rapid dynamics also put a constant and high demand on the system for controlling actin cable extension rates and lengths. How this regulation is achieved it not fully understood, but appears to involve actin turnover-promoting factors such as cofilin and Aip1 (Okada et al., 2006) and formin-binding proteins that modulate formin actin assembly activity.

Factors that promote yeast formin-mediated actin assembly include profilin, Bud6, Bil1, and Aip5 (Evangelista et al., 1997; Moseley et al., 2004; Graziano et al., 2013; Glomb et al., 2019; Xie et al., 2019). Bud6 directly interacts with G-actin and the C-terminal tail regions of Bni1 and Bnr1, positioning actin monomers near their actin-nucleating formin homology 2 (FH2) domains (Graziano et al., 2011). Interestingly, the Bud6-binding site (BBS) is positioned differently in Bni1 compared to Bnr1, leading to a key difference in their regulation. The BBS on Bni1 is adjacent to its C-terminal diaphanous autoregulatory domain (DAD), which is some distance from the FH2 domain (Moseley and Goode, 2005). As a result, Bud6 alone enhances Bni1-mediated actin nucleation. In contrast, the putative BBS on Bnr1 is much closer to its FH2 domain, and as a result, Bud6 alone obstructs actin nucleation by Bnr1 (Graziano et al., 2013). However, addition of Bil1, a small Bud6-binding protein, unmasks Bud6’s nucleation-promoting effects on Bnr1 (Graziano et al., 2013). Thus, Bil1 is required specifically for productive interactions of Bud6 with Bnr1, but not Bni1. In addition to factors that enhance nucleation, there are a number of yeast formin-binding partners that inhibit its nucleation and/or elongation activities, including Bud14, Smy1, and Hof1 (Chesarone et al., 2009; Chesarone-Cataldo et al., 2011; Graziano et al., 2013; Eskin et al., 2016; Garabedian et al., 2018). How these different stimulatory and inhibitory inputs work in concert to tune actin cable extension rate and length *in vivo* is only beginning to be understood.

All three known formin inhibitors in yeast (Hof1, Bud14, and Smy1) regulate Bnr1, but not Bni1. Whereas Bud14 and Smy1 inhibit Bnr1-mediated actin filament elongation (Chesarone et al., 2009; Chesarone-Cataldo et al., 2011; Eskin et al., 2016), Hof1 specifically inhibits Bnr1-mediated actin nucleation and has no effects on filament elongation (Graziano et al., 2014; Garabedian et al., 2018). Hof1 is stably anchored to the septin collar at the bud neck (Kamei et al., 1998; Vallen et al., 2000), similar to Bnr1, and deletion of *HOF1* results in excessive actin assembly, leading to defects in cable orientation and vesicle transport (Graziano et al., 2014; Garabedian et al., 2018, 2020). Hof1 inhibition of Bnr1 is overcome by the nucleation-promoting factor (NPF) Bud6, which is delivered on secretory vesicles to the bud neck (Garabedian et al., 2018). This regulatory scheme of combining a stationary inhibitor (Hof1) with a mobile activator (Bud6) is thought to establish a positive feedback loop for promoting Bnr1-mediated actin cable nucleation. Biochemical experiments have elucidated parts of the underlying mechanism. A C-terminal fragment of Bud6 (489–788), called ‘Bud6(L),’ enhances Bnr1-mediated actin nucleation when accompanied by its *in vivo* binding partner *Bud6 Interacting Ligand 1* (Bil1) (Graziano et al., 2013). These observations, supported by additional *in vivo* evidence, suggest that Bud6 and Bil1 work together to promote Bnr1-dependent actin cable nucleation.

In the present study, we identify YGL015c, a previously uncharacterized gene product, as a novel regulator of Bnr1 activity and cellular function. YGL015c encodes a 15 kDa protein, and in earlier proteomic studies was shown to interact with Bud6 and actin (Ito et al., 2001; Yu et al., 2008). This prompted us to investigate the potential role(s) of YGL015c in regulating formins and actin cable assembly. Based on its association with Bud6, and our own observations here of YGL015c co-localization with Bud6 on secretory vesicles, we named this gene *Bud6 Interacting Ligand 2* (*BIL2*). Our biochemical and genetic results suggest that Bil2/YGL015c functions as a novel inhibitor of Bnr1-mediated actin nucleation, but not elongation, and that it shares an essential *in vivo* function with the formin regulator Hof1.

## Materials and Methods

### Plasmids and Yeast Strains

A low-copy (CEN) plasmid was used to express GFP*-SEC4* in *S. cerevisiae* (Calero et al., 2003). A CEN plasmid for expressing GFP*^*Envy*^-SEC4* in *S. cerevisiae* was constructed by amplification of the GFP^*Envy*^ sequence from plasmid pFA6a-link-Envy-spHis5 (Slubowski et al., 2015) using primers (forward 5- CAT GCT GTC GAC ATG TCT AAA GGC GAG GAA TT-3; reverse 5- CAT TAG TCT AGA TTT GTA CAA TTC GTC CAT TC-3). The GFP^*Envy*^ sequence was then stitched in frame with *SEC4* using SalI and XbaI sites into the above-mentioned GFP-*SEC4* plasmid. A GFP*^*Envy*^-BIL2* plasmid was constructed by PCR amplification of the *YGL015c* open reading frame using primers (forward 5- CAT TAG TCT AGA ATG GAA GAC ACG ATA CGT CC-3; reverse 5- TAC GAT CCG CGG CTA ATC ATC AGA AGT GCA GC-3). This PCR product was cloned in frame with GFP^*Envy*^ using XbaI and SacII sites in the GFP*^*Envy*^-SEC4* plasmid above. Importantly, the sequence of the *YGL015c* open reading frame we amplified matched the sequence at the *Saccharomyces* Genome Database (SGD). A mCherry-*SEC4* plasmid was constructed by PCR amplification of the mCherry sequence using primers (forward 5-TAG TCA GTC GAC ATG GTG AGC AAG GGC GAG GA-3; reverse 5- TGC TAC TCT AGA TTA CTT GTA CAG CTC GTC CA-3). This product was cloned in frame with *SEC4*, as above, using SalI and XbaI sites. The plasmids used for galactose-inducible expression in *S. cerevisiae* of 6His-fusions of Bnr1 FH1-FH2-C (residues 757–1375) and Bnr1 FH2 (868–1291) have been described (Moseley and Goode, 2005; Okada et al., 2010; Jaiswal et al., 2013). A plasmid used for *Escherichia coli* expression of full-length 6His-Bil2 was constructed by PCR amplification of the *YGL015c* open reading frame using primers (forward 5-GAC TAG GGA TCC ATG GAA GAC ACG ATA CGT CC-3; reverse 5- TAG GAC AAG CTT CTA ATC ATC AGA AGT GCA GC-3). This PCR product was cloned in frame into the pET-28a vector using BamHI and HindIII sites. For expression of C-Bud6 in E. coli, the sequence encoding amino acids 489–788 of Bud6 was PCR amplified from genomic DNA and subcloned into pET-GST-TEV as previously described (Graziano et al., 2013).

All yeast strains used in this study are in the W303 background, with the exception of Figure 2E, which was in the ResGen background (see Supplementary Table 1). A *bil2*Δ strain (BGY4248) was generated by integration of a selectable marker into the *BIL2* locus as described (Longtine et al., 1998). To track secretory vesicles in live-imaging experiments, wildtype (BGY10) and *bil2*Δ (BGY4248) cells were transformed with pGFP*^*Envy*^-SEC4*. For analyzing Bnr1-GFP levels and neck localization, we crossed a Bnr1-GFP strain (BGY962) to a *bil2*Δ strain (BGY4248). For genetic analyses, we crossed a *bil2*Δ strain (BGY4248) to *hof1*Δ (BGY4253), *bud14*Δ (BGY4259), and *bud6*Δ (BGY1249). To localizing Bud6 and Bil2 *in vivo*, we used a Bud6-mCherry strain (BGY3913) (Garabedian et al., 2018) transformed with pGFP*^*Envy*^-BIL2*. For biochemical isolation of secretory vesicles, we used two different strains, one with differential tags on Bud6 and Bil2, and one with differential tags on Bil2 and Sec4, generated as follows. We crossed a *sec6-4* strain (a kind gift from Dr. Erfei Bi) to our *BUD6*-mCherry strain (BGY3913), producing *BUD6*-mCherry *sec6-4* (BGY4258), which was then transformed with pGFP*^*Envy*^-BIL2.* We transformed the same *sec6-4* strain with both pGFP*^*Envy*^-BIL2* and mCherry-*SEC4* plasmids.

### Protein Purification

Rabbit skeletal muscle actin was purified from acetone powder (Spudich and Watt, 1971) generated from frozen ground hind leg muscle tissue of young rabbits (Pel-Freez, United States). Lyophilized acetone powder stored at −80°C was mechanically sheared in a coffee grinder, resuspended in G-buffer [5 mM Tris-HCl pH 7.5, 0.5 mM Dithiothreitol (DTT), 0.2 mM ATP and 0.1 mM CaCl_2_], and then cleared by centrifugation for 20 min at 50,000 × *g*. Supernatant was collected and further filtered with Whatman paper. Actin was polymerized by addition of 2 mM MgCl_2_ and 50 mM NaCl to the filtrate and overnight incubation at 4°C with slow stirring. The next morning, NaCl powder was added to a final concentration of 0.6 M, and the mixture was stirred for another 30 min at 4°C. The F-actin was pelleted by centrifugation for 150 min at 120,000 × *g*, and the pellet was solubilized by dounce homogenization and dialyzed against G-buffer for 48 h at 4°C. Monomeric actin was then precleared by centrifugation at 435,000 × *g*, and loaded onto a S200 (16/60) gel-filtration column (GE Healthcare, United States) equilibrated in G-Buffer. Peak fractions were stored at 4°C.

To biotinylate actin on cysteine 374, an F-actin pellet as above was dounced and dialyzed against G-buffer lacking DTT. Monomeric actin was then polymerized by addition of an equal volume of 2× labeling buffer (50 mM imidazole pH 7.5, 200 mM KCl, 0.3 mM ATP, and 4 mM MgCl_2_). After 5 min, the actin was mixed with a fivefold molar excess of NHS-XX-Biotin (Merck KGaA, Germany) and incubated in the dark for 15 h at 4°C. The F-actin was pelleted as above, and the pellet was rinsed with G-buffer, then homogenized with a dounce, and dialyzed against G-buffer for 48 h at 4°C. Biotinylated monomeric actin was purified further on an S200 (16/60) gel-filtration column as above. Aliquots of biotin-actin were snap frozen in liquid N_2_ and stored at −80°C.

To label actin with Oregon Green (OG) on cysteine 374, an F-actin pellet as above was dounced and dialyzed against G-buffer lacking DTT. Monomeric actin was then polymerized by addition of an equal volume of 2× labeling buffer. After 5 min, the actin was mixed with a fivefold molar excess of OG-488 iodoacetamide (Invitrogen), resuspended in anhydrous dimethylformamide, and then incubated in the dark for 15 h at 4°C. The labeled F-actin was pelleted as above, and the pellet was rinsed with G-buffer, depolymerized by Dounce homogenization, dialyzed against G-buffer for 60 h at 4°C, and applied to an S200 (16/60) gel filtration column. Peak fractions were dialyzed for 15 h against G-buffer with 50% glycerol and stored at −20°C.

To label actin with pyrenyl-iodoacetamide on cysteine 374 (Cooper et al., 1984; Graziano et al., 2013), an F-actin pellet prepared as above was dialyzed against pyrene buffer (25 mM Tris-HCl, pH 7.5, 100 mM KCl, 0.3 mM ATP, and 2 mM MgSO_4_) for 4 h and then diluted with pyrene buffer to 1 mg/mL (23.8 μM). A 10-fold molar excess of pyrenyl-iodoacetamide was added, and the actin solution was incubated overnight at 4°C. The reaction was then centrifuged for 3 h at 4°C 150,000 × *g* in a Ti60 rotor (Beckman Coulter, Indianapolis, IN, United States) to pellet the F-actin. F-actin pellets were dounced, and dialyzed against G-buffer for 48 h to depolymerize the actin, then loaded on a S200 (16/60) column equilibrated in G-buffer. Peak fractions were pooled, aliquoted, snap frozen in liquid N_2_, and stored at −80°C.

C-Bnr1 (FH1-FH2-C; 758-1375) and Bnr1-FH2 polypeptides (869–1,289) were expressed as N-terminal 6His-fusions in *S. cerevisiae* strain BJ2168 from high copy plasmids under the control of a galactose-inducible promoter (Moseley and Goode, 2006). For each purification, 2 L of yeast cells were grown in synthetic medium lacking uracil with 2% raffinose to OD_600_ = 0.6–0.9. Then expression was induced by addition of dry ingredients: 10 g yeast extract, 20 g peptone, and galactose (2% wt/vol). Cells were grown for 12–16 h at 30°C, then pelleted, washed in H_2_O, frozen dropwise in liquid N_2_, and stored at −80°C. To initiate a protein preparation, frozen yeast pellets were lysed mechanically in a coffee grinder cooled with liquid N_2_. Then, 20 g of lysed yeast powder was resuspended in 20 mL of buffer A (20 mM NaPO_4_, pH 7.4, 150 mM NaCl, 30 mM imidazole, 0.5 mM DTT, 1% NP-40) supplemented with protease inhibitor cocktail as above, and cleared by ultracentrifugation at 200,000 × *g* for 20 min in a TLA100.3 rotor (Beckman Coulter). Cleared lysates were then passed through a 0.45 μm syringe filter (Millex, MilliporeSigma; Darmstadt, Germany) and the 6His-tagged Bnr1 polypeptides were incubated for 1 h at 4°C with 2 mL of Ni-NTA beads (New England Biolabs; Ipswich, MA, United States) with gentle agitation. Beads were washed three times with 10 mL wash buffer (20 mM Tris pH 8.0, 500 mM NaCl, 0.5 mM DTT, and 30 mM Imidazole), and Bnr1 polypeptides were eluted with 4 mL of elution buffer (20 mM Tris pH 8.0, 500 mM NaCl, 0.5 mM DTT, and 300 mM Imidazole). Peak fractions were pooled and loaded on a PD10 desalting column (GE Life Sciences; Marlborough, MA, United States) equilibrated with HEKG_10_D buffer (20 mM HEPES, pH 7.5, 1 mM EDTA, 50 mM KCl, 10% [vol/vol] glycerol, and 1 mM DTT), then concentrated to ∼200 μL, aliquoted, snap frozen in liquid N_2_, and stored at −80°C.

*S. cerevisiae* profilin was expressed in BL21(DE3) *E. coli* and purified as described (Graziano et al., 2013). Bacterial cells were grown in terrific broth to log phase and induced with 0.4 mM IPTG for 3–4 h at 37°C. Cells were pelleted and stored at −80°C. Frozen pellets were thawed, resuspended in lysis buffer (20 mM Tris-HCl, pH 8.0) supplemented with a protease inhibitor cocktail (1 mM PMSF, 0.5 μM each of pepstatin A, antipain, leupeptin, aprotinin, and chymostatin), and lysed by incubation with lysozyme and sonication. Lysates were cleared by centrifugation at 200,000 × *g* at 4°C for 20 min in a TLA-100.3 rotor (Beckman Coulter). The supernatant was then loaded on a 5 ml HiTrap Q fast flow column (GE Healthcare), and profilin was eluted using a 75 mL salt gradient (0–400 mM NaCl) in 20 mM Tris-HCl, pH 8.0. Peak fractions were pooled, concentrated to 5 mL, and loaded on a Superdex (26/60) gel filtration column (GE Healthcare) equilibrated in G-buffer. Peak fractions were pooled, aliquoted, snap frozen in liquid N_2_, and stored at −80°C.

6His-Bil2 protein was expressed in Rosetta 2 BL21(DE3) *E. coli* cells carrying a plasmid for expression of rare codons (MilliporeSigma; Darmstadt, Germany). Cells were grown to OD_600_ = 0.7–0.9 in terrific broth supplemented with kanamycin and chloramphenicol to maintain selection of the expression plasmid and the pRARE plasmid, respectively. Expression was induced with 0.4 mM IPTG overnight at 18°C, and then cells were pelleted and stored at −80°C. To initiate a preparation, a cell pellet was thawed, resuspended in lysis buffer (20 mM Tris pH 8.0, 500 mM NaCl, 0.5 mM DTT, and 30 mM Imidazole) with the same protease inhibitor cocktail as above, and lysed by treatment with 1 mg/mL lysozyme, 0.1 mg/mL DNAse I, and sonication. Lysates were cleared by centrifugation at 10,000 × *g* for 20 min in an F21S-8 × 50y rotor (Thermo Fisher Scientific; Waltham, MA, United States), and the supernatant was mixed with 1 mL of Ni-NTA beads (New England Biolabs) and rotated at 4°C for 1 h. The beads were then washed 3 times with 10 ml wash buffer (20 mM Tris pH 8.0, 500 mM NaCl, 0.5 mM DTT, and 30 mM Imidazole) in a gravity column at 4°C. 6His-Bil2 was eluted from the beads using elution buffer (20 mM Tris pH 8.0, 500 mM NaCl, 0.5 mM DTT, and 300 mM Imidazole), exchanged into HEKG_10_D on a PD10 desalting column (GE Life Sciences), concentrated to ∼200 μL, aliquoted, snap frozen in liquid N_2_ and stored at −80°C.

Bud6(L) was expressed in BL21(DE3) *E. coli* and purified as previously described (Graziano et al., 2011). Bacterial cells were grown in terrific broth to late log phase and induced using 0.4 mM IPTG for 3–4 h at 37°C. Cells were pelleted and frozen at −80°C. Frozen pellets were thawed, resuspended in lysis buffer (50 mM Tris, pH 8.5, 150 mM NaCl, 5 mM EDTA, 1.5% sarkosyl, 5 mM DTT, and standard protease inhibitors), treated with lysozyme, and sonicated. Cell lysates were cleared by centrifugation at 12,000 rpm for 10 min in a Sorvall S600 rotor (Thermo Fisher Scientific). Triton X-100 (final concentration 3.3% [vol/vol]) was added to the supernatant, and the mixture was mixed with 1 ml of preswollen glutathione agarose in PBS (137 mM NaCl, 2.7 mM KCl, 4.3 mM Na2HPO4, and 1.47 mM KH2PO4, pH 7.4). After incubation at 4°C for 3–4 h, beads were washed four times with PBS, and then twice with HEKD (20 mM Hepes, pH 7.5, 1 mM EDTA, 50 mM KCl, and 1 mM DTT). Bud6(L) was cleaved from GST and released from beads by digestion with TEV protease for 2 h at room temperature and snap frozen.

### Fixed Cell Imaging

To analyze actin cable organization in cells, yeast were grown to OD_600_ = 0.4–0.6 in YEPD media, fixed in 5% formaldehyde for 45 min at room temperature, and then washed three times with PBS buffer. Cells were stained 1–3 days with Alexa Fluor 488 phalloidin (Life Technologies; Grand Island, NY, United States), and then washed three times with PBS buffer. For experiments in which actin cables were analyzed using SOAX, an open source program for biopolymer networks (Xu et al., 2015), cells were treated with 100 μM CK666 (Sigma-Aldrich; St. Louis, MO, United States) for 20 min before fixation to inhibit the Arp2/3 complex and remove cortical actin patches. Fixed and stained cells then were imaged by structured illumination microscopy (SIM) on a Nikon Ti-2 SIM-E inverted microscope with a Hamamatsu Orca Flash 4.0 camera controlled by NIS-Elements software (Nikon Instruments), using an exposure time of 200 ms at 488 nm excitation and 40% laser power. From the SIM images, individual cells were cropped and background was subtracted, then actin cables were analyzed using SOAX. To optimize detection of cables in the SOAX analysis, default settings were used, with two exceptions: R-threshold value was set to 0.008, and k-stretch factor was set to 0.5. This automated analysis quantifies number of cable segments in cells.

For coefficient of variation (CoV) analysis of cable distribution, samples of the cell preparations made above (fixed and phalloidin stained) were imaged by SIM, using the same settings as above. CoV measurements are made by first tracing the outline of the mother cell compartment in ImageJ, and then measuring the mean fluorescence of actin cable staining and the standard deviation. The CoV is a ratio of the standard deviation over the mean. Wildtype cells typically have brightly stained cables against a dark background, yielding a high standard deviation, and therefore a higher CoV. Mutants with disorganized and dispersed actin cable networks have lower stand deviation values and consequently lower CoVs.

### Live Cell Imaging

For imaging secretory vesicle traffic, wildtype and mutant yeast strains were transformed with a CEN plasmid expressing GFP^*Envy*^-Sec4. Cells were grown at 25°C to OD_600_ = 0.4–0.8 in synthetic selective media, then mounted on slides and imaged on an i-E upright confocal microscope (Nikon Instruments) with a CSU-W1 spinning disk head (Yokogawa), 100× oil objective (NA 1.4; Nikon Instruments), and an Ixon 897 Ultra-CCD camera (Andor Technology) controlled by NIS-Elements software (Nikon Instruments). Exposure times of 50 ms at 50% intensity (excitation 488 nm) were used to image cells for 2 min. Movies were analyzed in ImageJ as follows. Secretory vesicle movements were monitored within the mother cells (*n* > 25) of each strain by manually tracking the positions over time for 3–8 puncta (GFP^*Envy*^-Sec4) per cell. Tortuosity measurements were made by dividing the length of the path (from the initial point of movement to the bud neck) by the distance between the point of origin and the bud neck. In addition, for each strain, we calculated the fraction of GFP^*Envy*^-Sec4 puncta in the mother cell that were successfully transported to the bud during a 30 s observation window (*n* > 20 cells per condition).

To compare Bnr1-GFP (endogenously tagged) levels at the bud neck of wildtype and *bil2*Δ cells, yeast were grown at 25°C in synthetic media to OD_600_ = 0.4–0.8, then mounted on slides, and immediately imaged on an i-E upright confocal microscope (Nikon Instruments) with a CSU-W1 spinning disk head (Yokogawa), 100× oil objective (NA 1.4; Nikon Instruments), and an Ixon 897 Ultra-CCD camera (Andor Technology) controlled by NIS-Elements software (Nikon Instruments). Each image was acquired using an exposure time of 100 ms at 488 nm excitation with 20% laser power. Z-stacks (15 images) were obtained by capturing images every 0.2 μm, and were analyzed in ImageJ as follows; *Z*-stacks were combined using the “sum projection” function, then a box of fixed dimensions was drawn to encompass the bud neck and measure the Bnr1-GFP integrated fluorescence density.

For the Pearson’s correlation analysis of Bud6-mCherry and GFP^*Envy*^-Bil2 colocalization, yeast cells were grown in synthetic selective media to mid-log phase, mounted on slides, and immediately imaged as above. Images were acquired using exposure times of 200 ms for Bud6-mCherry (561 nm excitation; 40% laser power) and 500 ms for GFP^*Envy*^-Bil2 (488 nm excitation; 80% laser power). Individual cells from the images were cropped, background was subtracted, and colocalization was analyzed using the Coloc2 plugin in ImageJ.

To quantify the ratio of GFP-Sec4 signal in the bud versus the entire cell, yeast transformed with the low copy GFP-Sec4 plasmid were grown in synthetic selective media to mid-log phase, mounted on slides, and immediately imaged as above. Images were acquired using exposure times of 50 ms (488 nm excitation; 40% laser power). Integrated density values were determined by selecting the bud and the whole cell using the ROI tool in ImageJ. The ratio of the signal in these two compartments was calculated by dividing the amount of signal in the bud by the amount of signal in the whole cell.

### Secretory Vesicle Isolation

Secretory vesicles were isolated from yeast cells using methods adapted from those previously described (Harsay and Bretscher, 1995). Cells were grown at 25°C to OD_600_ = 0.7 in 1 L cultures of selective media, then shifted to 37°C for 2 h. Cells were then harvested by centrifugation for 20 min at 3,000 × *g*, washed with ice-cold 10 mM NaN_3_, and incubated for 15 min on ice in softening buffer [0.1 M Tris-H_2_SO_4_ (pH 9.4), 50 mM 1,3-mercaptoethanol, and 10 mM NaN_3_]. Next, cells were washed in ice-cold spheroplast wash buffer (1.4 M sorbitol, 50 mM KPi, pH 7.5, and 10 mM NaN_3_), resuspended in the same buffer supplemented with 0.15 mg/mL Zymolyase-100T (US Biological; Salem, MA, United States), and incubated at 37°C for 2 h with gentle agitation. Spheroplasted cells were harvested by gentle centrifugation at 2,000 × *g*, and then gently washed two times with ice-cold spheroplast wash buffer (no zymolase), resting the samples on ice for 10 min between each wash to allow pellets to loosen. Next, using a plastic transfer pipette, the pellets were gently resuspended (to minimize lysis) in 30 mL of lysis buffer (0.8 M sorbitol, 10 mM triethanolamine, 1 mM EDTA, adjusted to pH 7.2 using acetic acid, 1 mM PMSF, and 0.5 μM each of pepstatin A, antipain, leupeptin, aprotinin, and chymostatin). The spheroplasts were transferred to a tight-fitting glass Dounce homogenizer, lysed with 20 strokes of the pestle, and then centrifuged at 700 × *g* for 10 min, generating the P1 (pellet) and S1 (supernatant) fractions. Then, the S1 fraction was centrifuged at 13,000 × *g* for 20 min to generate the P2 fraction (containing large organelles) and the S2 fraction (containing secretory vesicles and soluble proteins). The S2 fraction was then centrifuged for 1 h at 100,000 × *g* to generate the P3 fraction (secretory vesicles) and S3 fraction (soluble proteins). The P3 fraction was resuspended in lysis buffer and mounted on slides, and imaged on an i-E upright confocal microscope (Nikon Instruments) with a CSU-W1 spinning disk head (Yokogawa), 100× oil objective (NA 1.4; Nikon Instruments), and an Ixon 897 Ultra-CCD camera (Andor Technology) controlled by NIS-Elements software (Nikon Instruments). Images were acquired using exposure times of 200 ms for GFP-Bil2 (488 nm excitation; 50% laser power) and 100 ms for mCherry-Sec4 and Bud6-mCherry (561 nm excitation; 50% laser power).

### Pyrene-Actin Assembly Assays

Gel-filtered monomeric actin (5% pyrene-labeled) in G-buffer (5 mM Tris-HCl pH 8.0, 0.2 mM ATP, 0.2 mM CaCl2, and 0.2 mM DTT) was converted to Mg-ATP-actin immediately before each reaction (Moseley and Goode, 2005). Final reactions were 60 μL containing 2 μM G-actin. To initiate a reaction, 42 μL of the ATP-G-actin stock was mixed with 15 μL of proteins and/or control HEKG_5_ buffer, then mixed with 3 μL of 20× initiation mix (40 mM MgCl_2_, 10 mM ATP, and 1 M KCl) to initiate polymerization. Fluorescence was monitored at excitation 365 nm and emission 407 nm at 25°C in a fluorimeter (Photon Technology International, Lawrenceville, NJ, United States).

### Total Internal Reflection Fluorescence (TIRF) Microscopy

Glass coverslips (60 mm × 3 mm × 24 mm; Thermo Fisher Scientific) were cleaned by sonication for 30 min in detergent, followed by 1 M KOH, and 1 M HCl, and then stored in 100% ethanol. Coverslips were coated with a mixture of 4 mg/mL polyethylene glycol (PEG)-silane and 80 mg/mL biotin-PEG in 80% ethanol pH 1.0, then washed with water and dried with compressed N2. PEG-coated coverslips were stored for 1–3 days at 70°C prior to use. Flow chambers were constructed by sandwiching glass coverslips on top of plastic flow chambers (Ibidi, Fitchburg, WI, United States) using double-sided tape (2.5 cm 3 mm × 2 mm × 3 mm × 120 mm) and 5-min epoxy resin (Devcon, Riviera Beach, FL, United States). To anchor actin filaments in TIRF reactions, 4 mg/mL streptavidin in HEK buffer (20 mM HEPES pH 7.4, 1 mM EDTA, and 50 mM KCl) was flowed into the TIRF chamber for 15 s using a syringe pump (Harvard Apparatus, Holliston, MA, United States). Then the chamber was washed with HEK buffer + 1% BSA. The chamber was then equilibrated with TIRF buffer [10 mM HEPES pH 7.4, 50 mM KCl, 1 mM MgCl2, 1 mM EGTA, 0.2 mM ATP, 10 mM DTT, 15 mM glucose, 20 mg/mL catalase, 100 mg/mL glucose oxidase, 10 mM Imidazole, and 0.5% methylcellulose (4000 cP)]. Prior to experiments, the proteins used in TIRF reactions [His6-Bil2, Bud6(L), C-Bnr1, profilin, and Bnr1-FH2] were diluted into TIRF buffer. A fixed volume of proteins (different combinations) was rapidly mixed with a final concentration of 1 μM G-actin (10% OG-labeled, 0.2% biotinylated, as indicated) and flowed into the TIRF chamber. The TIRF chamber was then immediately mounted on the microscope for imaging. The delay between mixing proteins and initial imaging was 60 s. Time-lapse TIRF imaging was performed on a Ti200 inverted microscope (Nikon Instruments, New York, NY, United States) equipped with 100 mW solid-state lasers (Agilent Technologies, Santa Clara, CA, United States), a CFI Apo 60× 1.49 N.A. oil-immersion TIRF objective (Nikon Instruments), a iXon EMCCD camera with a pixel size of 0.267 mm (Andor Technology), and an additional 1.5× zoom module (Nikon Instruments). Focus was maintained using the Perfect Focus System (Nikon Instruments), and frames were captured every 10 s for a total of 600 s (10 ms at 488 nm excitation, 15% laser power) using NIS Elements software (Nikon Instruments). Image analysis was performed in FIJI, where background fluorescence was removed from each time series using the background subtraction tool in Fiji (rolling ball radius, 50 pixels). For measuring the number of actin filaments nucleated in TIRF reactions, fields of view were analyzed 200 s after the initiation of TIRF imaging. For each TIRF reaction, four separate fields of view were monitored and analyzed. To measure elongation rates, filament lengths were measured at different time points (using the freehand line tool in ImageJ). This analysis was limited to filaments that could be tracked for at least 60 s without growing out of the field of view. To measure filament elongation rates, plots of filament length versus time were generated, and the rates of elongation were determined from the slopes of the lines. To express rates in actin subunits s^–1^, we used the conversion factor of 374 subunits per μm length of F-actin (Huxley and Brown, 1967).

## Results

### Bil2 Is Required for Proper Transport of Post-Golgi Secretory Vesicles From the Mother Cell to the Bud

**FIGURE 1:**
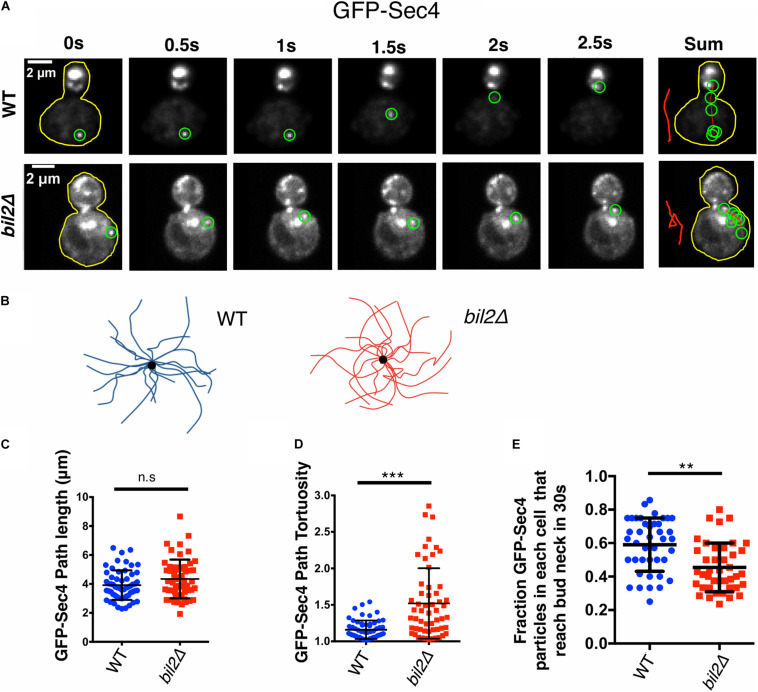
*BIL2* is required for efficient polarized delivery of secretory vesicles. **(A)** Representative time-lapse images showing the transport path of a secretory vesicle (GFP^*Envy*^-Sec4) moving from the mother to the bud. At each time point shown, the vesicle being tracked is highlighted by a green circle. To the right is a sum of vesicle positions over time, with a line (red) marking the transport path. The sum of the transport paths are isolated and expanded on the right. **(B)** Bouquets of representative transport paths for secretory vesicles (15 each) in wildtype and *bil2Δ* cells. Vesicle traces are organized such that they start at the periphery of the bouquets and terminate at the central dot (corresponding to the bud neck). **(C)** Quantification of GFP^*Envy*^-Sec4 path lengths from traces as in **(B)** (*n* = 25 vesicles per experiment, 2 independent experiments. Number of cells: experiment 1: 17 for each strain; experiment 2: 18 for each strain). **(D)** Tortuosity of transport paths (ratio of path length to distance traveled) for the same vesicles in **(C)**. **(E)** Fraction of vesicles successfully transported from the mother compartment to the bud during a 30 s observation window (*n* = 20 cells per condition per experiment, 2 independent experiments). Each dot represents the fraction of vesicles successfully transported to the bud in one cell. In all panels, bars show mean and SD. Statistical significance calculated by 2-way student *T*-test in all panels (n.s., no significance, *p ≤ 0.05, **p ≤ 0.01, ***p ≤ 0.001).

There are no known defects in cell growth or morphology caused by a deletion of YGL015c (henceforth referred to as *BIL2*), and we confirmed this here. However, a genome-wide study reported that *bil2*Δ cells abnormally accumulate a cargo protein in the *trans*-Golgi, suggesting a defect late in the secretory pathway (Proszynski et al., 2005). We considered the possibility that this defect might arise from altered actin organization, given that post-Golgi vesicles are transported on actin cables to the bud. Therefore, we used live imaging to compare the spatial distribution and movements of secretory vesicles (marked with GFP-Sec4) in wildtype and *bil2*Δ cells (Figure 1A). Overall, vesicles were polarized to the bud to a similar degree in wildtype and *bil2*Δ cells (Supplementary Figures 1A,B). However, our analysis of vesicle movements revealed differences in *bil2*Δ cells. To analyze the vesicle movements, we traced their paths of transport over time (example traces in Figure 1B), and then quantified path lengths and tortuosity (ratio of path length to distance traveled). Vesicle paths in *bil2*Δ cells were not significantly longer compared to wildtype cells (Figure 1C), but changed direction more often, making them circuitous (Figure 1D). We also assessed the overall efficiency of vesicle traffic, by quantifying the fraction of vesicles in mother cells that successfully translocated to the bud compartment during a 30 s window, and observed a modest yet significant decrease in transport efficiency in *bil2*Δ cells (Figure 1E). Together, these observations show that *bil2*Δ cells are partially defective in transporting post-Golgi vesicles to the bud, which may explain the previously observed cargo buildup in the *trans*-Golgi of *bil2*Δ cells (Proszynski et al., 2005).

### Loss of BIL2 Leads to Disorganized Actin Cable Networks Assembled by Bnr1

**FIGURE 2:**
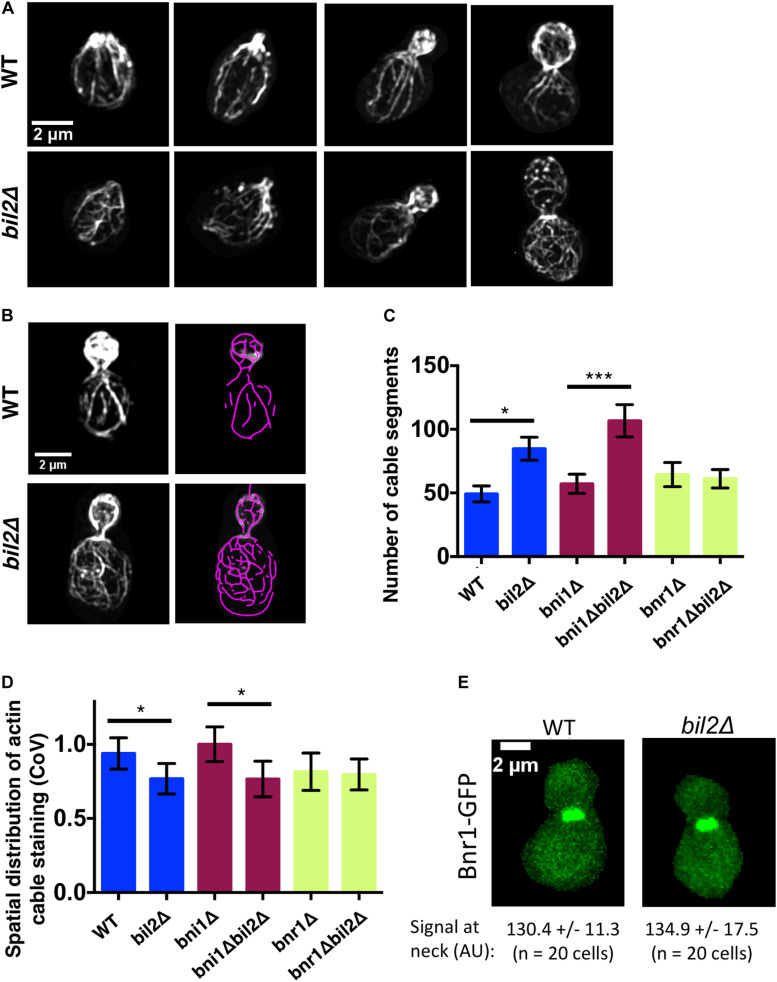
Loss of *BIL2* disrupts the spatial organization of Bnr1-polymerized actin cable networks. **(A)** Representative structured illumination microscopy (SIM) images of F-actin organization in CK666 treated, phalloidin stained wildtype and *bil2*Δ cells at different stages of bud growth. **(B)** Automated traces of actin cables from SIM images as in **(A)**, created using SOAX. Left, phalloidin stained cell. Right, purple cable segments generated by SOAX. **(C)** Average number of actin cable segments per cell analyzed by SOAX (*n* = 20 cells per condition from two independent experiments). **(D)** Coefficient of variation (CoV) of phalloidin staining within the mother compartment of cells treated with CK666 (*n* = 20 cells per condition from two independent experiments). **(E)** Representative images of endogenously-expressed Bnr1-GFP in wildtype (WT) and *bil2*Δ cells, with quantification of signals at the bud neck (mean and SD) below each image. In all panels, statistical significance calculated by 2-way student *T*-test (n.s., no significance, *p ≤ 0.05, **p ≤ 0.01, ***p ≤ 0.001).

Previously, we showed that mutants in *smy1* and *hof1* that have defective actin cable organization also show altered vesicle path lengths and tortuosity (Eskin et al., 2016; Garabedian et al., 2018). Therefore, the circuitous nature of the vesicle paths in *bil2Δ* cells prompted us to carefully compare cable organization between wildtype and *bil2Δ* cells using super-resolution structured illumination microscopy (SIM). Loss of *BIL2* led to a visible disorganization of cable networks, with minimal effect on polarized distribution of cortical actin patches (Figure 2A). To analyze actin cable organization defects in a more quantitative and unbiased manner, we also employed an open source program (SOAX), which skeletalizes the cable networks from cell images (Xu et al., 2015). Cells were pretreated with the Arp2/3 complex inhibitor CK666 to remove actin patches before this analysis to provide a less obstructed view of the cable networks and increase the accuracy of the SOAX analysis (Figure 2B). We focused our analysis on the cable networks in the mother cells, and found that *bil2Δ* cells have an increased number of cable segments compared to wildtype cells (Figure 2C). Additionally, we performed coefficient of variation (CoV) analysis on the same mother cells, measuring the mean fluorescence of cable staining and dividing by the standard deviation of the fluorescence (Figure 2D). Since wildtype cells have a well-defined and brightly stained set of actin cables against a dark background, they have a relatively high standard deviation, and a higher CoV. In contrast, mutants with disorganized cable networks, e.g., *hof1*Δ cells (Garabedian et al., 2020), have a lower standard deviation, and a lower CoV. Our data show that *bil2*Δ cells have a lower CoV compared to wildtype cells, which agrees with our SOAX analysis, and together these results indicate that Bil2 is required for the formation of properly organized actin cable networks.

We next asked whether *BIL2* contributes to the organization of actin cables assembled by Bni1 and/or Bnr1, which grow from the bud tip and bud neck, respectively. A comparison of cable organization in *bni1*Δ and *bni1*Δ*bil2*Δ cells, and in *bnr1*Δ and *bnr1*Δ*bil2*Δ revealed that the loss of *BIL2* significantly impaired actin cable organization in the *bni1*Δ background, but no the *bnr1*Δ background. These results suggest that *BIL2* functions to regulate *BNR1*-nediated actin assembly to govern proper cable organization in the mother cell (Figures 2C,D). Further, the loss of *BIL2* showed no effect on Bnr1-GFP levels at the bud neck (Figure 2E), indicating that Bil2 does not influence cable architecture by changing Bnr1 protein levels or localization.

### Bil2 Inhibits Bnr1-Mediated Actin Nucleation *in vitro*

**FIGURE 3:**
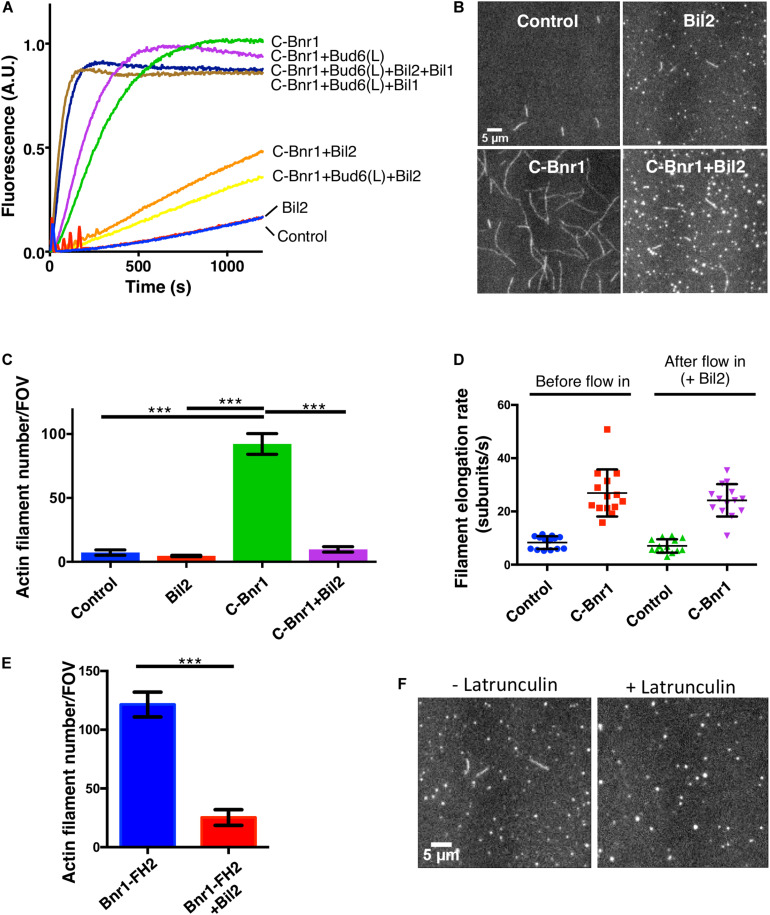
Purified Bil2 inhibits Bnr1-mediated actin nucleation but not elongation. **(A)** Bulk pyrene-actin assembly assays showing that Bil2 inhibits Bnr1-dependent actin nucleation, both in the presence and absence of Bud6. Bil2 with Bud6 and Bil1 present did not inhibit Bnr1. Reactions contain 2 μM actin monomers (5% pyrene labeled) and 5 μM profilin, with 2 nM C-Bnr1 (FH1-FH2-C; 758–1,375), 100 nM Bud6(L) (489–788), 100 nM Bil1, and/or 100 nM Bil2, as indicated. **(B)** Representative images from TIRF microscopy experiments showing the effects of Bil2 on Bnr1-mediated actin assembly. Reactions contain 1 μM actin monomers (10% Oregon green labeled) and 3 μM profilin, with 0.1 nM C-Bnr1 and/or 100 nM Bil2, as indicated. Images shown are from 200 s after the initiation of actin assembly. **(C)** Quantification of the number of actin filaments nucleated per field of view (FOV) at 200 s into TIRF reactions as in **(B)** (four FOVs per condition). Shown are the mean and SEMs. **(D)** Quantification of filament elongation rates for TIRF reactions as in **(B)**, except that 100 nM Bil2 was flowed into reactions 5 min after initiation of actin assembly (*n* = 20 filaments per condition). **(E)** Bil2 inhibits Bnr1 (FH2)-mediated actin filament assembly. Quantification of number of filaments nucleated per field of view (FOV) at 200 s into TIRF reactions as in B (four FOVs per condition). Reactions contain 1 μM actin monomers (10% Oregon green labeled) and 0.1 nM Bnr1 (FH2), with and without 100 nM Bil2. **(F)** TIRF fields showing that Bil2 produces latrunculin-resistant actin puncta. Reactions contain 1 μM actin monomers (10% Oregon green labeled), 3 μM profilin, and 100 nM Bil2, with or without 100 nM Latrunculin B. Images shown are from 200 s after the initiation of actin assembly. Shown are the mean and SEMs. Statistical significance calculated by 2-way student *T*-test in all panels (n.s., no significance, *p ≤ 0.05, **p ≤ 0.01, ***p ≤ 0.001).

Our *in vivo* observations above inspired us to test *in vitro* whether Bil2 has any effects on Bnr1-mediated actin assembly activity. To address this, we purified 6His-Bil2 from *E. coli* and first tested its effects in bulk actin assembly assays. As expected, C-Bnr1 (FH1-FH2-C) rapidly nucleated actin polymerization (Figure 3A), and was enhanced by its nucleation-promoting factors Bud6(L) and Bil1 (Graziano et al., 2013). The addition of Bil2 strongly inhibited C-Bnr1 effects, both in the presence and absence of Bud6(L), but had no effect on the assembly of actin alone in the absence of C-Bnr1. Interestingly, however, the further addition of Bil1 to reactions containing Bil2, Bud6(L), and C-Bnr1 led to rapid actin assembly. On the other hand, Bil1 failed to release C-Bnr1 from Bil2 inhibition in the absence of Bud6(L) (Supplementary Figure 2). Thus, Bil1 and Bud6(L) together are required to overcome Bil2 inhibition of C-Bnr1.

To gain additional insights into Bil2 inhibitory effects on C-Bnr1, we used TIRF microscopy assays, and directly visualized individual actin filaments being assembled in real time, where we could distinguish effects on nucleation from effects on filament elongation. In these assays, C-Bnr1 alone increased the number of new filaments formed compared to control reactions, and Bil2 inhibited the nucleation effects (Figures 3B,C). To assess whether Bil2 also affects the rate of filament elongation, we pre-assembled filaments in the presence or absence of C-Bnr1, and then flowed in Bil2 or control buffer, and monitored change in filament length over time. As expected, C-Bnr1 markedly increased the rate of filament elongation in the presence of profilin (Chesarone-Cataldo et al., 2011). Flowing in Bil2 did not significantly alter the rate of filament elongation by C-Bnr1 (Figure 3D), suggesting that Bil2 acts on C-Bnr1 primarily to inhibit actin nucleation and not elongation.

To better understand how Bil2 blocks Bnr1-mediated actin nucleation, we asked whether it can inhibit an FH2 domain-only (Bnr1-FH2) construct. These nucleation assays were performed in the absence of profilin, since FH2 domains (without FH1 domains) nucleate actin assembly only in the absence of profilin (Sagot et al., 2002b). Bil2 strongly inhibited Bnr1-FH2 nucleation activity (Figure 3E), suggesting that it may interact with the FH2 domain to block nucleation.

Finally, in our TIRF experiments, we noticed that all Bil2-containing reactions had a number of small puncta (marked by labeled actin), regardless of whether or not those reactions contained C-Bnr1 (Figure 3B). Therefore, we asked whether the puncta were comprised of F-actin or G-actin by pre-incubating reactions with Latrunculin B to block actin polymerization (Coué et al., 1987). While Latrunculin B blocked actin filament formation, as expected, it did not block formation of the actin puncta induced by the presence of Bil2 (Figure 3F). These observations suggest that Bil2 may bind to actin monomers, consistent with its reported two-hybrid interaction with actin (Yu et al., 2008).

### BIL2 and HOF1 Genetically Interact and Share an Essential *in vivo* Function

**FIGURE 4:**
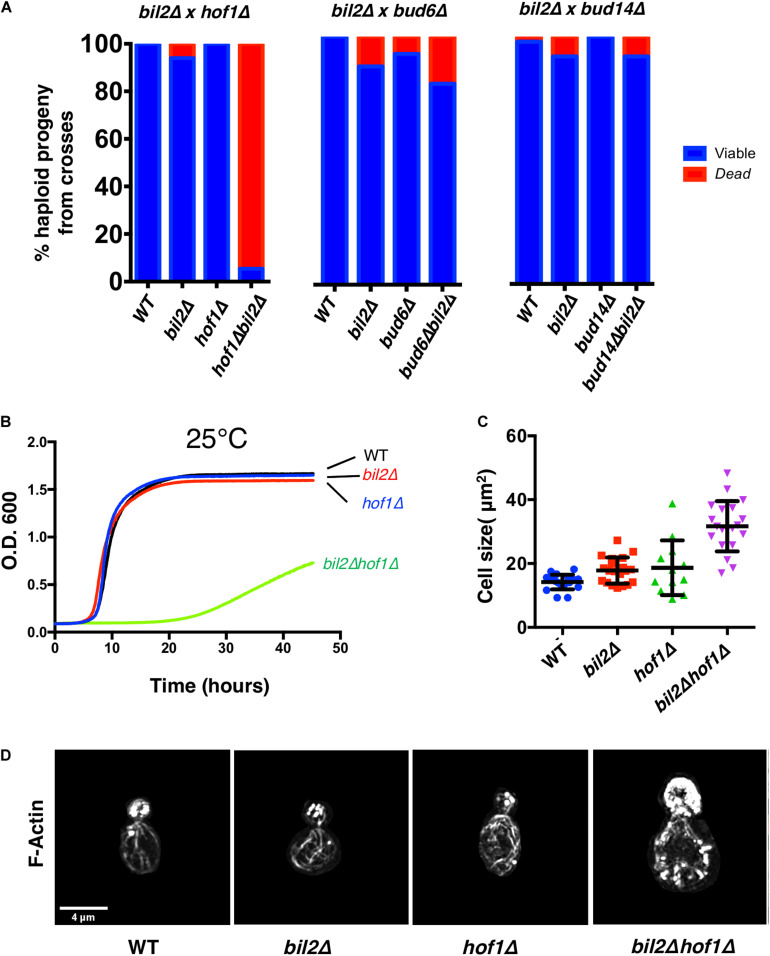
Synthetic genetic interactions between *BIL2* and *HOF1*. **(A)** *bil2*Δ haploids were crossed to haploids carrying deletions in other Bnr1 regulatory genes, including *HOF1*, *BUD6*, and *BUD14*. Diploids were sporulated, and tetrads dissected and genotyped (*n* = 144, 64, and 104 tetrads from crosses with *hof1*Δ, *bud6*Δ, and *bud14*Δ, respectively). Resulting wildtype, single mutant, and double mutant haploids were analyzed for viability at 25°C. **(B)** The indicated haploid strains were compared for growth in synthetic complete media at 25°C in a shaking microplate reader for 50 h, monitoring growth (OD_600_) every 5 min. Lines represent the average of three independent cultures per strain. **(C)** Cell size was determined from DIC images in ImageJ by outlining each cell and calculating its area (*n* = 20 cells per condition). Shown are the mean and SD. **(D)** Representative max projection *Z*-stacks of phalloidin stained cells imaged by structured illumination microscopy (SIM). Note cell size is to scale, i.e., *hof1*Δ bil2Δ cells are enlarged compared to wildtype, *hof1*Δ, and *bil2*Δ cells, as indicated in **(C)**.

The activity profile of Bil2, as an inhibitor of Bnr1-mediated actin nucleation without affecting elongation, is similar to only one other known yeast formin regulator, Hof1. This prompted us test genetic interactions between *BIL2* and *HOF1*. We therefore crossed *bil2*Δ and *hof1*Δ haploid strains, and as controls crossed *bil2*Δ to mutants in two other yeast formin regulators, *bud6*Δ and *bud14*Δ. The resulting diploids were sporulated, tetrads were dissected, and haploid progeny were assessed for growth. This analysis revealed that the majority of *bil2*Δ*hof1*Δ double mutants were inviable, as compared to control crosses where the majority of double mutants were viable (Figure 4A). These observations demonstrate that *BIL2* and *HOF1* share an essential function *in vivo*. To gain additional insights into this function, we analyzed the viable *bil2*Δ*hof1*Δ double mutants. Compared to single mutants, the viable double mutants were severely compromised for cell growth (Figure 4B) and had enlarged cell sizes (Figure 4C) and disorganized actin cable networks (Figures 4C,D). Together, these *in vivo* observations suggest that Bil2 and Hof1 may perform related, complementary roles in controlling Bnr1-mediated actin cable nucleation and polarized cell growth.

### Bil2 Localizes to Polarity Sites and Associates With Secretory Vesicles

**FIGURE 5:**
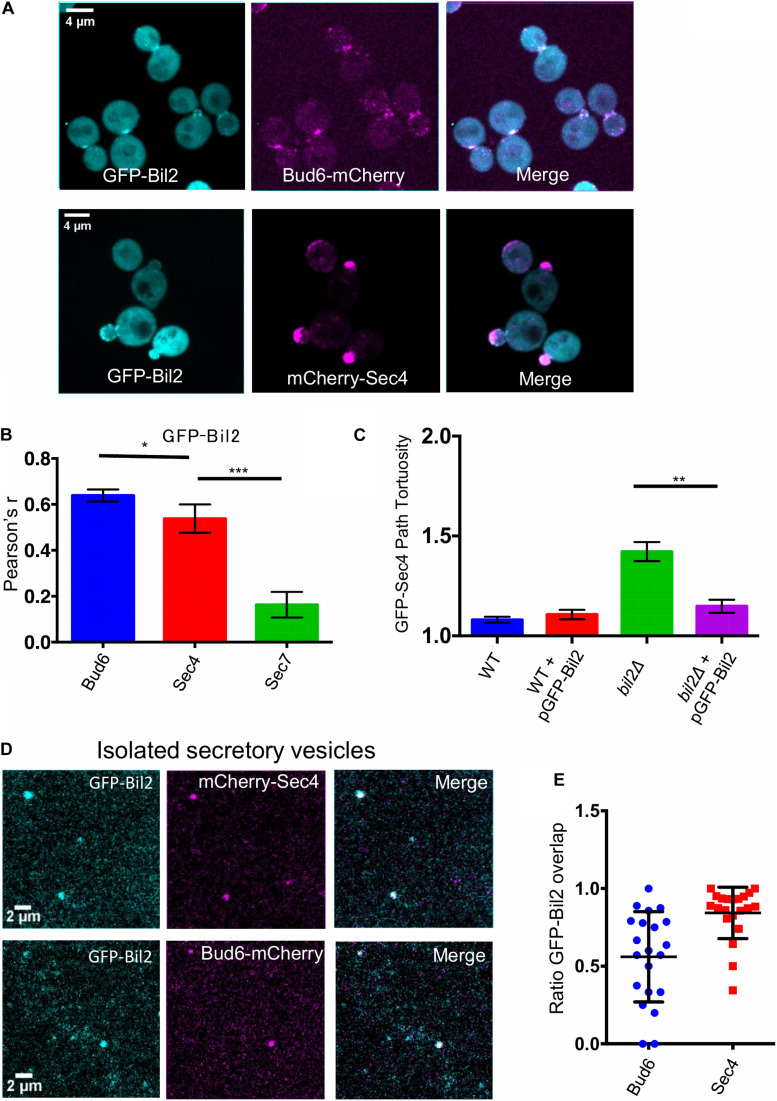
GFP-Bil2 localization to polarity sites and association with secretory vesicles. **(A)** Representative images of live cells expressing GFP-Bil2 (from a low copy plasmid under the control of the *ACT1* promoter) and either integrated Bud6-mCherry or mCherry-Sec4 (expressed from a low copy plasmid under the control of its own promoter). **(B)** GFP-Bil2 colocalization in live cells with mCherry-Sec4 (secretory vesicle marker) or Sec7-mCherry (*trans*-Golgi marker) quantified by Pearson correlation. **(C)** Comparison of GFP-Sec4 vesicle transport paths (ratio of path length to distance traveled) in wildtype (WT) and *bil2*Δ cells with or without the pACT1-GFP-Bil2 plasmid (*n* = 25 vesicles per condition). **(D)** Representative fields of view of secretory vesicles isolated from cells expressing GFP-Bil2 (plasmid, as in **A**) along with Bud6-mCherry (integrated) or mCherry-Sec4 (plasmid, as in **A**). **(E)** Quantification of GFP-Bil2 colocalization with Bud6- and Sec4-positive secretory vesicles. Statistical significance in all panels calculated by 2-way student *T*-test (n.s., no significance, *p ≤ 0.05, **p ≤ 0.01, ***p ≤ 0.001).

To gain additional insights into Bil2 *in vivo* function, we investigated the localization of Bil2 endogenously tagged at its C-terminus with GFP or 3GFP. Unfortunately, we could not detect the expression of endogenously tagged Bil2-GFP or Bil2-3GFP. It is not clear whether Bil2 expression is very low to begin with, or the C-terminal tags reduced the level of expression. However, we were able to detect N-terminally tagged GFP-Bil2 expressed from a low copy plasmid under the control of the strong constitutive *ACT1* promoter (Figure 5A). Importantly, this plasmid complemented *bil2*Δ defects in secretory vesicle transport (Figure 5B), suggesting that although the protein is likely to be expressed at higher levels than endogenous Bil2, it is nonetheless capable of performing Bil2’s normal functions. GFP-Bil2 localized to the cytosol, to the bud neck and bud tip (sites of polarized growth), and to faint mobile puncta (suggestive of secretory vesicles). However, we acknowledge that addition of the GFP tag and/or the overexpression of Bil2 may alter its normal localization pattern. Interestingly, Bud6-GFP localizes to similar sites, although it shows more pronounced localization to polarity sites and secretory vesicles compared to Bil2 (Jin and Amberg, 2000; Segal et al., 2000). Deletion of *BUD6* did not noticeably change GFP-Bil2 localization (Supplementary Figure 3A), and deletion of *BIL2* did not noticeably change Bud6-GFP localization (Supplementary Figure 3B). Thus, despite their ability to interact, Bud6 and Bil2 appear to localize independently to polarity sites.

Localization of GFP-Bil2 in live cells overlapped significantly with Bud6-mCherry and with mCherry-Sec4 (Figure 5C). Bud6 has been localized to secretory vesicles (Garabedian et al., 2018), which have a similar appearance to the faint mobile puncta we observed for GFP-Bil2. To further test the association of Bil2 with secretory vesicles, we performed a biochemical fractionation. Differential centrifugation was used to isolate secretory vesicles from cells co-expressing GFP-Bil2 with either Bud6-mCherry or mCherry-Sec4, and then colocalization was assessed by microscopy (Figure 5D). The majority of GFP-Bil2 puncta (∼80%) colocalized with mCherry-Sec4, and approximately half of the GFP-Bil2 puncta colocalized with Bud6-mCherry (Figure 5E). These results more conclusively demonstrate that Bil2 associates with secretory vesicles, and suggest that approximately half of the Bil2-positive vesicles also harbor Bud6.

## Discussion

The initial goal of this study was to explore the cellular functions of a previously uncharacterized gene, YGL015c (*BIL2*), which was reported to interact with actin and the formin nucleation-promoting factor Bud6 (Ito et al., 2001; Yu et al., 2008). A previous proteomic screen had also identified this gene as being required for normal delivery of a marker protein to the cell surface via the secretory pathway, revealing that in *bil2*Δ cells the surface protein aberrantly accumulated in the *trans*-Golgi compartment (Proszynski et al., 2005). However, the specific role(s) of *BIL2* in this pathway were unclear. Given its suggested interactions with Bud6, which promotes formin-mediated actin cable nucleation, we decided to explore the possibility that Bil2 regulates formin-mediated actin cable assembly. Our *in vivo* observations showed that *bil2*Δ cells have defects in Bnr1-dependent actin cable architecture, including an increase in the total number of cable segments and a disorganization or entanglement of cable networks in mother cells. Consistent with these defects, the transport paths of secretory vesicles in *bil2*Δ cells were more circuitous compared to the paths of vesicles in wildtype cells. Further, purified Bil2 inhibited Bnr1-mediated actin nucleation but not filament elongation *in vitro*, both in bulk and TIRF microscopy assays. Based on these genetic and biochemical observations, we propose that Bil2 functions, at least in part, as a novel inhibitor of Bnr1-mediated actin cable nucleation required for proper secretory traffic.

Although a number of direct regulators of Bnr1 activity have been identified to date, the only other one with an activity profile similar to Bil2 is the F-BAR protein Hof1. Like Bil2, Hof1 inhibits Bnr1-mediated actin nucleation but not filament elongation (Graziano et al., 2014). In addition, both Bil2 and Hof1 inhibit the actin-nucleating FH2 domain of Bnr1. A low-resolution EM structure of the Hof1-FH2 complex revealed that the F-BAR domain of Hof1 binds to the FH2 domain near its actin-binding surfaces (Garabedian et al., 2018). It is possible that Bil2 uses a related mechanism to inhibit Bnr1. Alternatively, a Bil2-actin complex might directly interact with the Bnr1 FH2 domain to block nucleation. Indeed, it was recently shown that the mammalian formin INF2 is inhibited by binding of a cyclase-associated protein (CAP)-actin complex (Mu et al., 2019). Consistent with their related biochemical activities in suppressing Bnr1-mediated actin nucleation, we found that *bil2*Δ and *hof1*Δ mutations are synthetic lethal. These results suggest that Bil2 and Hof1 have overlapping, possibly complementary roles in controlling formin-mediated actin cable assembly *in vivo*. Nearly all of the *bil2*Δ*hof1*Δ double mutants were lethal, possibly due to a lethal level of disrupted secretory traffic and impaired polarized growth. The small percentage of *bil2*Δ*hof1*Δ double mutant cells that were viable grew very slowly and had enlarged cell morphologies.

Our observations raise the question of why yeast cells have so many different inhibitors for one formin (Bnr1). Bil2 and Hof1 inhibit Bnr1-mediated actin nucleation and genetically interact. Bud14 and Smy1 inhibit actin filament nucleation and elongation by Bnr1 and genetically interact (Chesarone et al., 2009; Chesarone-Cataldo et al., 2011). None of these four inhibitors of Bnr1 have any direct effects on Bni1 activity. Thus, cells appear to require tight spatiotemporal control over Bnr1 activity (nucleation and elongation) in order to build proper cable networks consisting of the appropriate number of cables with the appropriate length and architecture for optimal secretory traffic. It is also worth noting that no inhibitors of Bni1 activity have been identified to date. This may be related to Bnr1 having a ∼15-fold stronger nucleation activity compared to Bni1 (Moseley and Goode, 2005). In addition, it may be significant that Bnr1 is stably tethered to the bud neck, whereas Bni1 is dynamically recruited from the cytosol to the bud cortex, where it is transiently activated to nucleate cable assembly and then released (Buttery et al., 2007). These differences in the dynamics of the two formins may result in their activities requiring distinct regulatory mechanisms. Further, Bnr1 assembles cables that fill the mother cell, where cable overgrowth can be detrimental to secretory traffic. Thus, Bnr1 (but not Bni1) may require stronger inhibition to restrict its activity.

**FIGURE 6:**
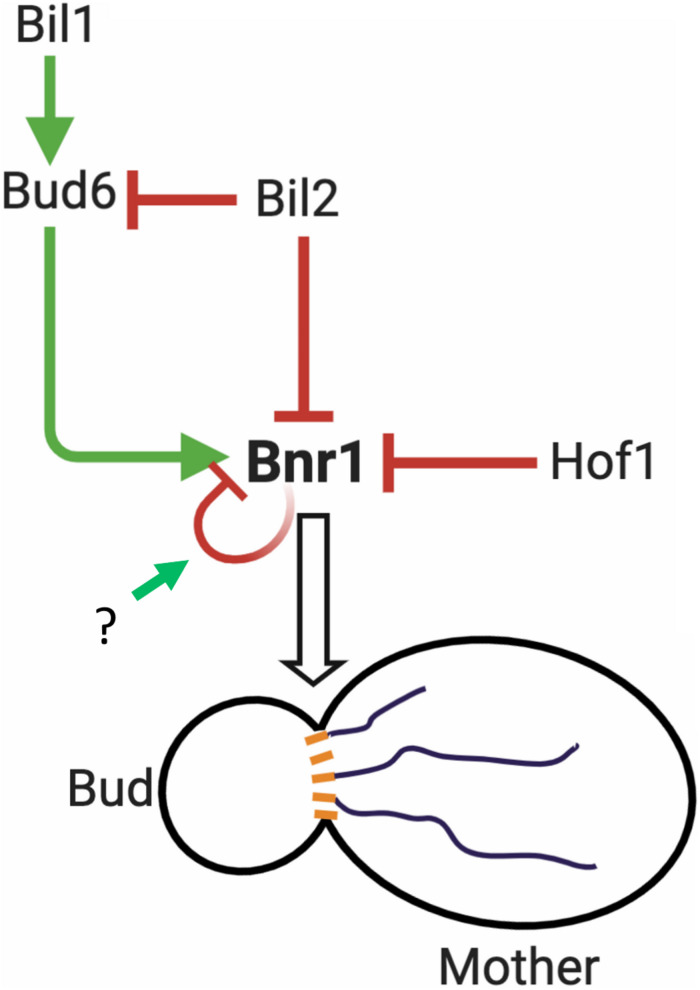
Working model for the regulation of Bnr1-mediated actin cable nucleation. Bnr1 is anchored at the bud neck and assembles cables in the mother cell. Bud6 functions as an NPF, promoting actin nucleation by Bnr1. Bil2 and Hof1 specifically inhibit actin nucleation by Bnr1. Bud6 is delivered on secretory vesicles to the bud neck and overcomes Hof1 inhibition of Bnr1 as part of a positive feedback loop promoting cable assembly (Garabedian et al., 2018). Bud6 is not sufficient to overcome Bil2 inhibition of Bnr1. However, bud6 together with its binding partner Bil1 overcomes Bil2 inhibition to promote Bnr1-mediated actin nucleation. Bil2 and Bud6 are found together on many secretory vesicles, suggesting that Bil2 may help keep Bud6 inactive until it reaches the bud neck where Bil1 is found (Graziano et al., 2013). Bnr1 is also predicted to be autoinhibited via interactions of its N-terminal diaphanous inhibitory domain (DID) with its C-terminal diaphanous autoregulatory domain (DAD) (Li et al., 2003). However, it is not yet clear what mechanisms trigger the release of Bnr1 from autoinhibition. After a filament is nucleated, Bnr1 remains processively attached to the growing barbed end, where the duration and rate of filament elongation are controlled by other cellular factors, including Smy1 and Bud14 (Eskin et al., 2016). Model created using BioRender.com.

How do cells overcome Bil2 and Hof1 inhibition of Bnr1-mediated actin nucleation In the case of Hof1, its inhibitory effects on Bnr1 are overcome by the formin NPF Bud6, which depends on direct binding of Bud6 to Bnr1 (Garabedian et al., 2018). *In vivo*, Hof1 is anchored at the bud neck where Bnr1 also resides, and Bud6 is delivered on secretory vesicles to the bud neck. Genetic and biochemical evidence suggest that upon arrival Bud6 triggers Bnr1’s release from Hof1 inhibition to promote actin cable assembly as part of a positive feedback loop. In contrast, we found that Bud6 alone is not sufficient to overcome the inhibitory effects of Bil2 on Bnr1. Instead, this requires Bud6 and its ligand Bil1. Thus, Bil1 appears to be specifically required for overcoming Bil2 inhibition of Bnr1, but not Hof1 inhibition of Bnr1. Similar to Bud6, we found that Bil2 associates with secretory vesicles. Thus, Bil2 may serve to inhibit Bud6’s NPF activity while on vesicles until it arrives to the bud neck, where Bil1 relieves inhibition. Importantly, our results do not rule out the possibility that Bil2 has additional functions (beyond directly regulating Bnr1 activity) that influence cable architecture and/or the transport of vesicles along cables. Indeed, the yeast formin inhibitor Smy1 not only directly regulates Bnr1 activity but also plays an important role in recruiting myosin to secretory vesicles *in vivo*, and increases myosin processivity *in vitro* (Hodges et al., 2009; Lwin et al., 2016). Collectively, these observations lay a foundation for understanding the *in vivo* regulatory circuit controlling Bnr1-mediated actin nucleation (Figure 6). However, they also raise many new questions that need to be answered in future studies, including: (i) when and where each regulatory protein interacts with Bnr1, and with each other, *in vivo*, (ii) whether their effects on Bnr1 are regulated by post-translational modification, and (iii) what mechanism(s) trigger the release of Bnr1 from autoinhibition.

Finally, it will be important to determine if and how Bil2 (and Bil1) influence the other known cellular functions of Bud6, particularly its role in microtubule plus end capture and mitotic spindle orientation (Segal et al., 2002). Bud6 binds not only to formins but also the microtubule plus end-binding protein EB1 (Bim1), and is believed to coordinate actin and microtubule functions *in vivo* (Delgehyr et al., 2008; Ten Hoopen et al., 2012). Therefore, it will interesting to learn whether Bil1 and/or Bil2 contribute to this cytoskeletal crosstalk by Bud6. Further, what we learn from studying Bil1, Bil2, and Bud6 in yeast may provide valuable lessons for understanding the mechanisms coordinating actin and microtubule functions in other systems. While there are no clear homologs of Bil1, Bil2, or Bud6 outside of the fungal kingdom, mounting evidence suggests that adenomatous polyposis coli (APC) protein is a functional counterpart to Bud6 in animal cells. Similar to Bud6, APC binds to EB1 and serves as a formin NPF *in vitro* and *in vivo* (Okada et al., 2010; Breitsprecher et al., 2012; Juanes et al., 2017, 2019). Further, APC interacts with a large number of other cytoskeletal regulatory proteins. Some of these ligands may regulate APC’s NPF activities in a manner related to how Bil1 and Bil2 regulate Bud6 NPF activity. Indeed, it was recently shown that EB1 directly inhibits the NPF activity of APC’s Basic domain (Juanes et al., 2020). These findings, together with the results presented here, suggest that evolutionarily diverse organisms may have adapted to use distinct sets of proteins (lacking obvious sequence homology) to establish common regulatory schemes for controlling actin assembly.

## Data Availability Statement

The original contributions generated for this study are included in the article/Supplementary Material, further inquiries can be directed to the corresponding author.

## Author Contributions

TR and BG designed the research and wrote the manuscript. TR carried out all of the experiments and data analysis. BG obtained funding and supervised the project. Both authors contributed to the article and approved the submitted version.

## Conflict of Interest

The authors declare that the research was conducted in the absence of any commercial or financial relationships that could be construed as a potential conflict of interest.
