# Supplementary material for: Bil2 Is a Novel Inhibitor of the Yeast Formin Bnr1 Required for Proper Actin Cable Organization and Polarized Secretion
Source: Front Cell Dev Biol. 2021 Feb 9;9:634587. doi: 10.3389/fcell.2021.634587 (PMC7900418; doi:10.3389/fcell.2021.634587)
Supplement: Supplementary file 1 [file Table_1.pdf]

Figure S1

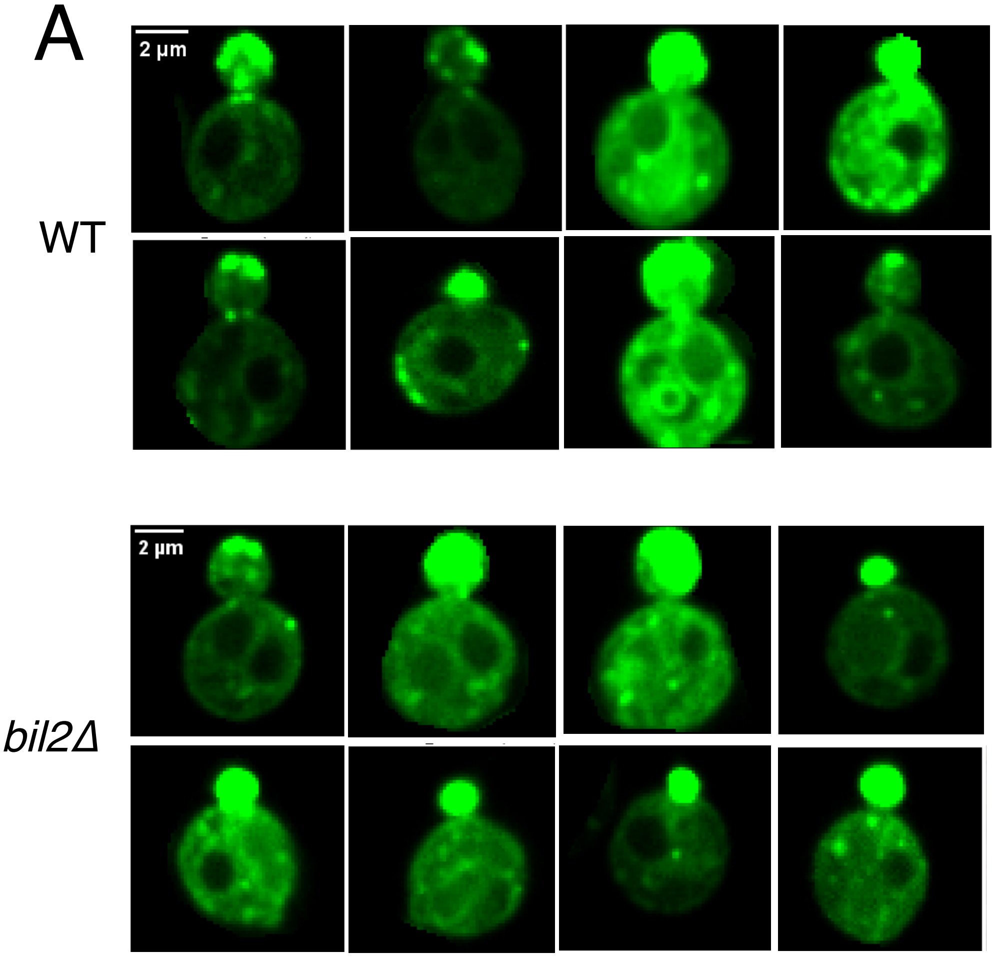

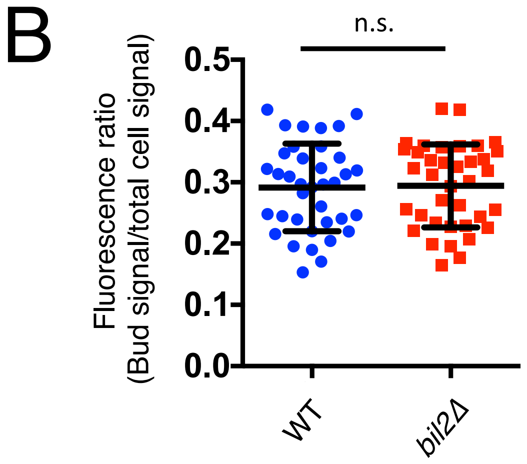

Figure S1. Polarized distribution of GFP-Sec4 signal in wildtype and *bil2*Δ cells. (A) Representative images of wildtype and *bil2*Δ cells expressing GFP-Sec4 from a low copy plasmid. Note the cell-to-cell variability in total GFP-Sec4 levels, which stems from differences in plasmid copy number. (B) Comparison of GFP-Sec4 polarized distribution (ratio of GFP signal in the bud divided by the total signal). Data from two independent experiments (n=37 cells per condition). Statistical significance calculated by 2-way student T-test (n.s., no significance).

Figure S2

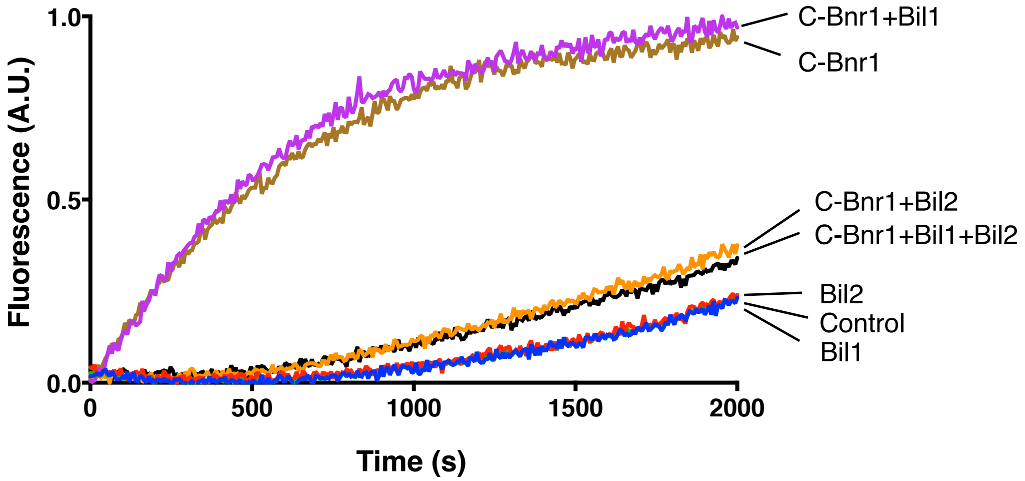

Figure S2. Control reactions accompanying the bulk actin assembly assays in Figure 3A. The data shown demonstrate that Bil2 inhibits C-Bnr1-mediated actin nucleation both in the presence and absence of Bil1. Reactions contain 2 μM actin monomers (5% pyrene-labeled) and 5 μM profilin, with 2 nM C-Bnr1 (FH1-FH2-C; 758-1375), 100 nM Bil1, and/or 100 nM Bil2, as indicated.

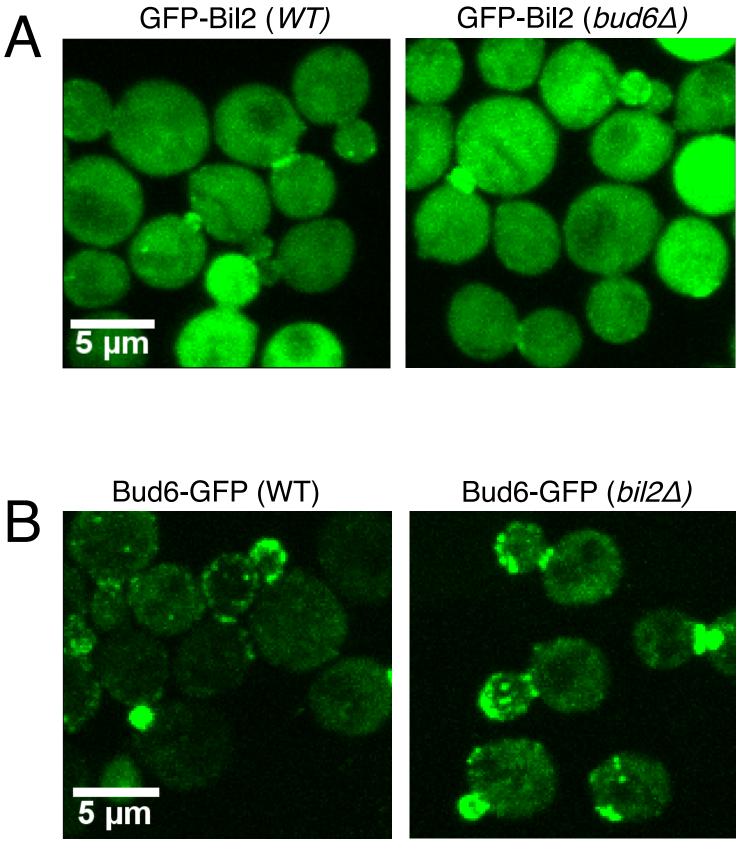

Figure S3. Bil2 and Bud6 localize independently to cell polarity sites. (A) Representative images of live wildtype (WT) and *bud6*Δ cells expressing GFP-Bil2 (from a low copy plasmid under control of the *ACT1* promoter). (B) Representative images of live wildtype (WT) and *bil2*Δ cells expressing endogenously tagged Bud6-GFP.

Table I. ***S. cerevisiae*** **strains used in this study.**

Name	Relevant genotype	Strain background
BGY10	*MAT* a, *his3-11,15;ura3-52;leu2-3,112;ade2-1;trp1-1;psi+*	w303
BGY4248	*MAT* a, *his3-11,15;ura3-52;leu2-3,112;ade2-1;trp1-1;psi+; bil2Δ::KanMX*	w303
BGY3239	*MAT* a, *his3-11,15;ura3-52;leu2-3,112;ade2-1;trp1-1;psi+; bni1Δ::TRP1*	w303
BGY3242	*MAT* a, *his3-11,15;ura3-52;leu2-3,112;ade2-1;trp1-1;psi+; bnr1Δ::HIS3mx6*	w303
BGY4249	*MAT* a, *his3-11,15;ura3-52;leu2-3,112;ade2-1;trp1-1;psi+; bnr1Δ::HIS3mx6; bil2Δ::KanMX*	w303
BGY4250	*MAT* a, *his3-11,15;ura3-52;leu2-3,112;ade2-1;trp1-1;psi+; bni1Δ::TRP1; bil2Δ::KanMX*	w303
BGY4251	*MAT* a, *ura3Δ0, leu2Δ0, his3Δ1, met15Δ0 BNR1-5xGly-GFP::HIS3mx6*	ResGen
BGY4252	*MAT* a, *ura3Δ0, leu2Δ0, his3Δ1, met15Δ0 BNR1-5xGly-GFP::HISmx6 bil2Δ::KanMX*	ResGen
BGY4253	*MAT* a, *his3-11,15;ura3-52;leu2-3,112;ade2-1;trp1-1;psi+; hof1Δ::HIS3mx6*	
BGY3181	*MAT* a, *his3-11,15;ura3-52;leu2-3,112;ade2-1;trp1-1;psi+; hof1Δ::HISmx6; bil2Δ::KanMX*	w303
BGY4254	*MAT* a, *his3-11,15;ura3-52;leu2-3,112;ade2-1;trp1-1;psi+; BUD6-mCherry::HIS3mx6*	w303
BGY1249	*MAT* a, *his3-11,15;ura3-52;leu2-3,112;ade2-1;trp1-1;psi+; bud6Δ::TRP1*	w303
BGY4255	*MAT a his3-11,15;ura3-52;leu2-3,112;ade2-1;trp1-1;psi+; BUD6-GFP::TRP1*	w303
BGY4256	*MAT* a, *his3-11,15;ura3-52;leu2-3,112;ade2-1;trp1-1;psi+ bil2Δ::KanMX; BUD6-GFP::TRP1*	w303
BGY4257	*MAT* a, *his3-11,15;ura3-52;leu2-3,112;ade2-1;trp1-1;psi+; sec6-4::TRP1*	w303
BGY4258	*MAT* a, *his3-11,15;ura3-52;leu2-3,112;ade2-1;trp1-1;psi+; sec6-4::TRP1; BUD6-mCherry::HISmx6*	w303
BGY4259	*MAT* a, *his3-11,15;ura3-52;leu2-3,112;ade2-1;trp1-1;psi+; bud14Δ::URA3*	w303
